# Correlation between coronavirus conspiracism and antisemitism: a cross-sectional study in the United Kingdom

**DOI:** 10.1038/s41598-023-41794-y

**Published:** 2023-12-05

**Authors:** Daniel Allington, David Hirsh, Louise Katz

**Affiliations:** 1 King’s College London, London, UK; 2 Goldsmiths, University of London, London, UK; 3 Xenophon College, London, UK

**Keywords:** Psychology, Human behaviour

## Abstract

This article presents the findings of a survey of UK-resident adults ($n$ = 1790) carried out in December 2021 and designed to test for a relationship between antisemitism and coronavirus conspiracism. Antisemitism was measured using the Generalised Antisemitism (GeAs) scale, and coronavirus conspiracism was measured using a version of the Flexible Inventory of Conspiracy Suspicions (FICS). Hypotheses and methodology were pre-registered, and all data and code are open. There was found to be a positive correlation between coronavirus conspiracy suspicions and Generalised Antisemitism, robust to demographic controls. This correlation appears to be entirely accounted for by older forms of antisemitism: antisemitism as expressed in relation to Israel and its supporters was found to be associated with coronavirus conspiracism only because both of these variables were associated with antisemitism as expressed in relation to Jews identified as Jews. Statistical analysis suggests that these findings may be generalised from the sample to the UK adult population with some confidence, although no data were collected in other national contexts, such that generalisation to other national contexts must remain speculative.

## Introduction

There has been a great deal of research into the relationship between medical conspiracy beliefs and attitudes and behaviours relating to infectious disease, especially with regard to health-protective measures such as vaccination^1–4^. As of 2022, nearly 100 studies were reported to have found a negative relationship between COVID-19 preventative behaviours and endorsement of misinformation and conspiracy theories with regard to the novel coronavirus responsible for COVID-19, i.e. SARS-CoV-19^5^. However, while many researchers have argued for a historical and contemporary relationship between antisemitism and other forms of conspiracy belief^6–18^, there has so far been no attempt to test the hypothesis of a statistical association between antisemitism and coronavirus conspiracism.

Although that hypothesis has not been tested as such, the possibility of such a relationship was assumed in one of the first British studies of coronavirus conspiracism, which found that ‘around one-fifth [of respondents] endorsed to some degree [the conspiracy theory] that “Jews have created the virus to collapse the economy for financial gain”^19^. This finding received wide coverage in the press^20–23^, but was immediately called into question by scholars who drew attention to methodological problems with the research, both in that the sample is likely to have been unrepresentative of the UK population, and in that a non-standard response scale was used. The first scholarly critique of the paper pointed out that the same study reported roughly similar proportions of respondents blaming Muslims, aliens, pharmaceutical corporations, 5G radiation, or Bill Gates for the pandemic, with even higher proportions attributing its rise to the machinations of the World Health Organisation, the United Nations, or the Chinese government, and suggested that the implausibility of these findings called into doubt the high rate of agreement also found for the antisemitic coronavirus conspiracy theory quoted above^24^. Thus it is unsurprising that the findings of a follow-up survey carried out by a different team suggested that the original study may have exaggerated true levels of agreement with all of the conspiracy theories that it fielded, both antisemitic and otherwise^25^.

Despite this, the idea of a relationship between antisemitism and coronavirus conspiracism has remained of political interest^26^, and a number of studies have provided qualitative evidence for the plausibility of such a relationship^17,27–30^. Moreover, one quantitative study found frequent blaming of Jews for the pandemic in a sample of Twitter posts about COVID-19^31^. Thus, the current study was designed in order to test for the possibility of a statistical association between antisemitism and coronavirus conspiracism. In so doing, it aims to contribute not only to knowledge on the subject of antisemitism and conspiracism, but also to knowledge on the broader relationship between prejudice and attitudes to the coronavirus, complementing studies of other forms of racism as manifested during the pandemic.

Although multiple forms of prejudice have been linked to the recent pandemic^32,33^, there are particular difficulties in the measurement of antisemitism specifically. It has been persuasively argued that, in the post-Holocaust era, antisemitism can be expressed not only in relation to Jews identified as Jews, but also in relation to Israel and its supporters^13,34–41^. While this principle has received some critique on a theoretical level^42^, it has received empirical support from all of the various studies which have been carried out to test the hypothesis of a correlation between attitudes to Jews *qua* Jews and attitudes to Israel and its supporters in multiple national contexts^18,43–51^. Recognition that ‘antizionist’ or Israel-focused expressions of antisemitism had been growing in political force^52^ led to the formulation of the International Holocaust Remembrance Working Definition of Antisemitism, generally referred to as the IHRA Definition^53^, which, as of 2020, had been adopted by 39 countries, with support also expressed by the United Nations, the Organisation of American States, and the European Council, Parliament, and Commission^54^. Although the precise details of the IHRA Definition are subject to debate (a point to which we return below), its primary contribution is to acknowledge that antizionist forms of antisemitism — or antisemitic forms of antizionism — exist and are important, to suggest ways in which such expressions are typically manifest, and to affirm that criticism of Israel similar to that which is directed against other states is not antisemitic: all points which are in accordance with the broad thrust of the arguments and findings of the research literature already cited.

Widespread political and institutional adoption of the IHRA Definition prompted the drafting and release of two further definitions: the Jerusalem Declaration on Antisemitism^55^, which is usually regarded as a direct rival to the IHRA Definition, and the Nexus Document^56^, which has been presented as a clarification to the IHRA Definition. Considerable academic controversy exists around these three definitions^57–63^. However, unanimity exists between all three with regard to the fundamental point that antisemitism may be expressed through statements about Israel and its supporters as well as through statements about Jews *qua* Jews: the Jerusalem Declaration states that ‘hostility to Israel could be an expression of an antisemitic animus’, notes that ‘portraying Israel as the ultimate evil or grossly exaggerating its actual influence can be a coded way of racialising and stigmatising Jews’, and provides five examples of attitudes towards or actions taken in relation to Israel which ‘on the face of it, are antisemitic’^55^, while the Nexus Document states that ‘Israel is a magnet for and a target of antisemitic behaviour’, asserts that ‘[i]t is antisemitic to promote myths, stereotypes or attitudes about Zionism and/or Israel that derive from and/or reinforce antisemitic accusations and tropes’, and likewise provides five examples of antisemitic attitudes towards or actions taken in relation to Israel^56^. Thus, it seems clear that, when searching for correlations between antisemitism and any other trait, researchers will be reflecting something approaching a consensus position if they measure endorsement of ‘new’, Israel-related, or antizionist forms of antisemitism alongside older forms of antisemitism focused on Jews identified as Jews — especially in view of the scholarly evidence cited above, which establishes that the fundamental insights underpinning all three definitions are congruent with the empirical reality of antisemitism as a social phenomenon. This is exactly what the current study does, using a validated measurement instrument whose psychometric properties are a matter of public record (see “Measures”, below).

In order to achieve full transparency, the hypotheses, the data collection methodology, and the confirmatory analytic methodology were all pre-registered. Where the standard scientific approach of null hypothesis significance testing is employed (as here), it has been argued that ‘inferences from pre-registered analyses will be more reproducible than … analyses that were not preregistered because the relation between the analysis choices and findings cannot be influenced by motivation, memory, or reasoning biases’^64^. Use of pre-registration in this case thus provides greater confidence that reported findings of confirmatory analysis are not spurious: an important consideration, given the academic controversy to which this study responds, and also given its political implications. However, the confirmatory analysis, which simply reports the findings of the hypothesis tests is preceded by tables of descriptive statistics for the dataset, and by a discussion of associations identified therein which might be considered to compromise the findings of confirmatory analysis. These associations are further probed in an exploratory analysis which follows on from the confirmatory analysis. Through this means, and through the use of straightforward, well-understood, and easily interpretable tests, we hope to provide a relatively conclusive answer to the question of whether or not a statistical association might have existed, at least in the UK. Moreover, our open publication of the study’s dataset will enable other researchers to re-interrogate these findings using alternative analytic methodologies, and also to test for other relationships of interest through secondary analysis. Although the social context has now changed considerably, with the end of coronavirus pandemic mitigation policies such as lockdowns and compulsory mask wearing in much of the world, the findings may additionally provide a baseline for comparison in future research on other forms of prejudice and conspiracism.

## Hypotheses

- *H1* A positive correlation between antisemitism in general and coronavirus conspiracism
- *H2* A positive correlation between antisemitism expressed in relation to Jews identified as Jews and coronavirus conspiracism
- *H3* A positive correlation between antisemitism expressed in relation to Israel and its supporters and coronavirus conspiracism

## Methods

### Measures

In order to avoid potential problems with the use of a new instrument to measure endorsement of coronavirus conspiracy theories, an existing, psychometrically-validated instrument was employed, i.e. the five-item Flexible Inventory of Conspiracy Suspicions or FICS^65^. This is a standard template which can easily be adapted to measure conspiracism on virtually any topic, and which had already been adapted to the specific case of coronavirus conspiracism in at least one peer-reviewed study^66^. The version of the inventory adopted in the current study (see Supplementary Information) was identical to that employed in the latter, and a respondent’s mean score across the items of that inventory is here referred to as his or her Coronavirus Conspiracy Suspicion (CCS).

Antisemitism was likewise measured using an established instrument, i.e. the Generalised Antisemitism or GeAs scale (see Supplementary Information), which is is founded on an understanding of contemporary antisemitism as finding expression both in attitudes towards Jews *qua* Jews and in attitudes towards Israel and its supporters. The development of the Generalised Antisemitism scale and the rationale behind each of its 12 items has been fully documented in a peer-reviewed article^67^, and its factor structure and psychometric validity have been analysed in a separate peer-reviewed article^51^. An advantage of the Generalised Antisemitism scale is that it can be used to provide separate indices of ‘new’ and ‘old’ antisemitism, or a combined index representing the overlap between the two. As in other published studies employing this scale, a mean was therefore taken across all items of the scale to produce a single measure of Generalised Antisemitism (GeAs), and separate means were also taken across items of the two subscales to produce twin measures of Judeophobic Antisemitism (JpAs) and Antizionist Antisemitism (AzAs).

Ideology and support for political parties has been found to be relevant to some forms of conspiracy belief^68^. For the purposes of the current study, ideological position was measured using YouGov’s standard seven-point self-placement scale from ‘Very left wing’ to ‘Very right wing’. It is acknowledged that self-placement on such a scale is a crude measure of ideology, but it is widely used in studies both of antisemitism^69^ and of conspiracism^68,70^. In order to facilitate comparison with other studies, the entire seven-point ideological scale was retained for the purposes of descriptive analysis, although it could not be incorporated in the main analyses as its ordinal nature requires the calculation of a different coefficient of correlation. In addition, voting history was recorded with regard to the December 2019 UK general election, behaviour during which was recoded as a set of four dummy variables, one each for the two main parties (i.e. the right-wing Conservative Party and the left-wing Labour Party), one for all other parties, and one for not having voted. Correlations for these variables are reported in the ‘Sample descriptive statistics’ section, which reports descriptive statistics for the sample, with the ‘Exploratory analysis’ section being primarily reserved for exploration of a relationship (or, rather, lack of an expected relationship) which transpired to be more interesting, while also investigating the possible influence of mutual correlations with the most strongly correlated political or ideological variable.

### Data collection

Data were collected by YouGov from a sample of British adults randomly sampled from a recruited panel. As per YouGov’s standard practice, random sampling was done within quotas for greater demographic representativeness. Data collection was carried out online over the weekend beginning on the evening of Friday 17 December 2021. Data were not accessible by any human until the morning of Monday 20 December, and were electronically delivered to the researcher later that day. The registration document was submitted on Sunday 19 December. Thus, registration was carried out prior to any human observation of the data. Because the questionnaire was fielded as part of an omnibus survey, the sample size was not subject to change at the researchers’ request. Altogether, 1790 responses were collected.

### Missing data

With regard to all variables, skipped, refused, and ‘don’t know’ responses were treated as missing data, and were excluded from the mean when scoring Likert measures for each respondent. Where a respondent answered ‘don’t know’ for every item in a single scale or subscale, data for the measure in question were treated as missing. Data for voting history were recorded as missing for all respondents under the age of 20, regardless of how they answered the question, as it was impossible for a respondent aged under 20 at the time of data collection to have taken part in the UK General Election of 2019.

Of the observations collected, 1742 were complete with regard to Coronavirus Conspiracy Suspicion and Generalised Antisemitism, and 1270 were complete with regard to these variables as well as to the full set of demographic variables to be employed in the confirmatory analysis (see Sections “Confirmatory analytic methodology” and “Confirmatory analysis”, below). Ideological position was a missing variable for 392 respondents, with the result that there were only 1006 complete observations for one of the calculations carried out in the exploratory analysis (see Section “Exploratory analysis”, below).

### Confirmatory analytic methodology

All hypotheses were tested first by calculating the product-moment coefficient of correlation (popularly known as Pearson’s coefficient of correlation), and then by calculating the partial product-moment coefficient of correlation with controls for demographic variables (age, gender, ethnicity, and level of education). Correlations were calculated both before and after removal of outliers (defined as observations for which the Cook’s distance for the linear relationship between the dependent and independent variables is greater than three times the mean). For purposes of controls, educational level was operationalised as a dummy variable (degree/non-degree), as was gender (female/not female), and ethnicity (white/other-than-white). Hypotheses were tested through calculation of *p* values (two-tailed), with a pre-registered cut off of *p* < 0.010. 95% confidence intervals were reported in all cases. The pre-registered approach was to report findings for all hypotheses both before and after removal of outliers, although there did not transpire to be any outliers on the definition employed here.

### Power analysis

Given a cutoff of *p* < 0.010 and 1742 complete observations for Generalised Antisemitism and Coronavirus Conspiracy Suspicion, an $r$ of 0.10, i.e. the canonical cut-off for a reportable effect in analyses of correlation ^71^, can be detected with 95% power in a two-tailed test. Thus, it was unlikely that a non-negligible bivariate correlation in the general population would fail to be detected in the sample.

### Ethical approval and informed consent

All methods were performed in accordance with the relevant guidelines and regulations. Ethical clearance was sought and received from the Research Ethics Committee at King’s College London, MRM-20/21–20,918. Informed consent was obtained from all participants.

## Findings

### Sample descriptive statistics

See Table 1 for a full breakdown of the sample by demographic variables, ideology, and voting history. The means and percentages in the table are to be treated as descriptive of the sample rather than as the basis for inference about the population from which the sample was drawn; as such, no confidence intervals or significance tests are provided. Respondents are divided by age at the median for the sample, i.e. 49, and both ethnicity and education are disaggregated further than in the confirmatory analysis, with white British respondents being differentiated from white respondents of other-than-British backgrounds, and with educational level also being opertionalised on a tripartite scheme, such that, among those respondents not educated to degree level (referred to here as ‘high’ educational level), those without formal qualifications or whose formal qualifications are considered equivalent to the standard UK qualifications for age 16 school-leavers (referred to here as ‘low’ educational level) were distinguished from those with intermediate levels of education (referred to here as ‘medium’ educational level). Please note that respondents were given the option to identify their gender otherwise than as male or female, but none chose to do so. It may be observed that younger respondents tended to be more highly educated than older respondents, and that members of non-white ethnic groups tended to be younger and more highly educated than white British respondents, but that the most highly-educated ethnic category consisted of non-British white respondents. It is noted that respondents who did not vote in 2019 contained a higher proportion of white members of other-than-British origin (unsurprisingly, since citizens of European Union member states are not eligible to vote in British general elections unless they also hold British citizenship).

**Table 1:**
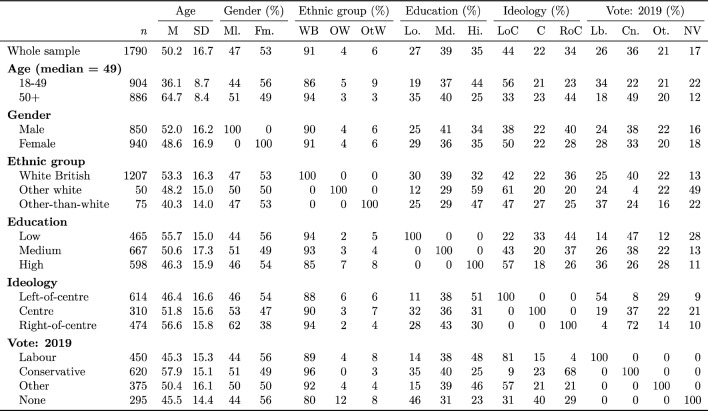
Breakdown of sample by demographic variables, self-declared ideological position, and voting history.

*Lb* Labour, *Cn* Conservative, *Ot* Other, *NV* Non-voter, *LoC* Left-of-centre, *C* Centre, *RoC* Right-of-centre, *Lo* Low, *Md* Medium, *Hi* High, *WB* white British, *OW* other white, *OtW* Other-than-white, *Ml* Male, *Fm* Female.

For Coronavirus Conspiracy Suspicion, Generalised Antisemitism, Judeophobic Antisemitism, and Antizionist Antisemitism by demographic variables, self-declared ideological position, and voting history, see Table 2. No attempt at statistical inference is made, in order to avoid exacerbating the multiple comparisons problem, correction for which would reduce statistical power for the confirmatory analysis. It may be observed that male respondents received higher scores for Judeophobic Antisemitism but lower scores for Antizionist Antisemitism than female respondents, and that respondents of median age or below received higher scores for Generalised Antisemitism and Antizionist Antisemitism, but very slightly lower scores for Judeophobic Antisemitism, than respondents of above-median age. It may also be observed that respondents from other-than-white ethnic groups received higher scores for Generalised Antisemitism, Judeophobic Antisemitism, Antizionist Antisemitism, and Coronavirus Conspiracy Suspicion, while less highly-educated respondents received higher scores for Generalised Antisemitism, Judeophobic Antisemitism, and Coronavirus Conspiracy Suspicion. There was nothing to suggest a relationship between level of education and Antizionist Antisemitism.

**Table 2:**
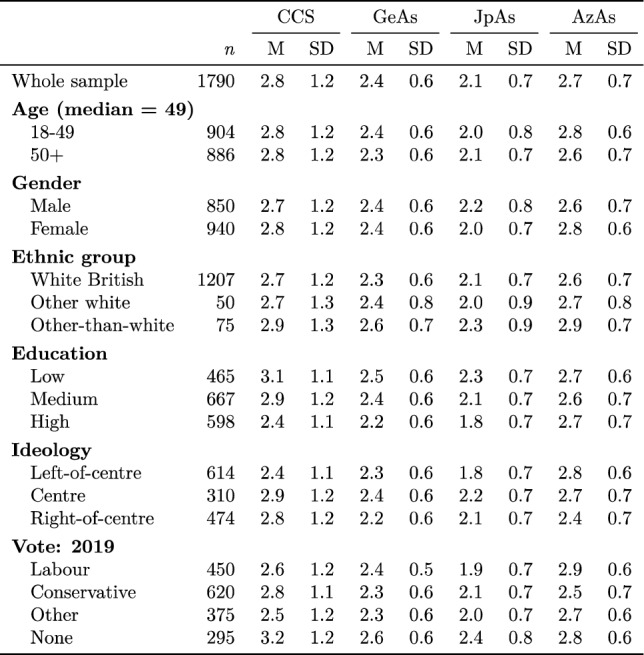
Pre-registered dependent and independent variables by demographic variables, self-declared ideological position, and voting history.

Respondents identifying with the centre and right of the ideological spectrum had higher mean scores for Coronavirus Conspiracy Suspicion and Judeophobic Antisemitism than respondents identifying the the left of the ideological spectrum, while respondents identifying with the left and centre of the ideological spectrum had higher mean scores for Antizionist Antisemitism and Generalised Antisemitism (which is the overlap between Judeophobic Antisemitism and Antizionist Antisemitism). Respondents who voted for Labour Party candidates and for candidates representing parties other than the Labour and Conservative Parties in 2019 had lower mean scores for Coronavirus Conspiracy Suspicion and Judeophobic Antisemitism (although the difference from those who voted for Conservative Party candidates was very slight with regard to the latter), while respondents who did not vote at all had the highest mean scores for Coronavirus Conspiracism, Generalised Antisemitism, and Judeophobic Antisemitism, and the second-highest mean score for Antizionist Antisemitism, with regard to which, Labour voters had slightly higher mean scores.

For correlations between all variables used in hypothesis testing, as well as demographic variables and dummy variables representing ideological position and voting history, see Table 3. No attempt at statistical inference is again made, for the reasons discussed above. It is noted that, while apparent relationships between ethnicity, Coronavirus Conspiracy Suspicion, and all forms of antisemitism were noted with regard to Table 2, the correlations are weak, especially with regard to Coronavirus Conspiracy Suspicion, where the correlation is so weak as to be treated as no correlation at all (although ethnicity was operationalised differently, with two categories rather than three). Given previous research (see above), the positive correlation between Judeophobic Antisemitism and Antizionist antisemitism was expected — a point to which we shall return below. For comparison, this positive correlation between ‘old’ and ‘new’ forms of antisemitism was equal in strength to the correlation between considering oneself to fall on the right of the ideological spectrum and not having voted Labour in 2019.

**Table 3:**
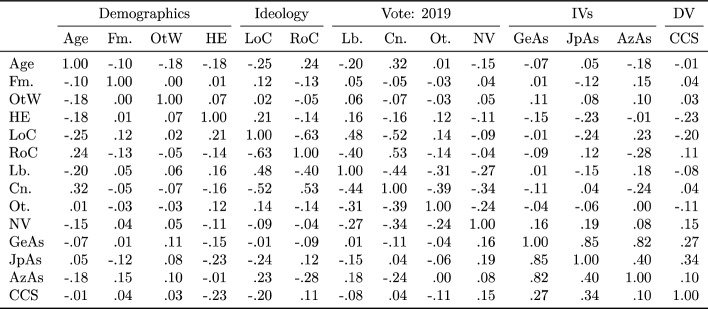
Correlation matrix.

*Lb* Labour, *Cn* Conservative, *Ot* other, *NV* non-voter, *LoC* left-of-centre, *RoC* right-of-centre, *Fm* female, *OtW* other-than-white, *HE* high education, *IVs* pre-registered independent variables, *DV* Pre-registered dependent variable.

From the point of view of this study’s pre-registered hypotheses, the most notable correlations were the positive correlations between Judeophobic Antisemitism and Coronavirus Conspiracy Suspicion and between Generalised Antisemitism and Coronavirus Conspiracy Suspicion (see Section “Confirmatory analysis”). These were stronger than the negative correlations between degree-level education and both Judeophobic Antisemitism and Coronavirus Conspiracy Suspicion. Correlations between the study’s hypothetical dependent and independent variables and the various dummy variables representing voting history and self-declared ideological position were for the most part weak or non-existent, with the exception of the roughly equal although opposite correlations between Judeophobic and Antizionist Antisemitism and declared position on the left of the ideological spectrum, the negative correlation between Antizionist Antisemitism and both declared position on the right of the ideological spectrum and a 2019 vote for the Conservative Party, and the negative correlation between Coronavirus Conspiracy Suspicion and self-placement on the left of the ideological spectrum. This correlation was stronger than the correlations between Coronavirus Conspiracy Suspicion and all of the dummy variables representing voting history (of which those representing votes for the two main parties were so weak as to be non-reportable).

Treating ideological self-identification as an ordinal variable (very left-wing = lowest, very right-wing = highest) and calculating the rank-order coefficient of correlation (popularly known as Spearman’s coefficient of correlation) was found to make little difference: in the sample, Coronavirus Conspiracy Suspicion is only weakly correlated with ideological position when operationalised in this way, *r*_s_ = 0.18, and is uncorrelated with Generialised Antisemitism, *r*_s_ =  − 0.03, having a moderate positive correlation with Judeophobic Antisemitism, *r*_s_ = 0.22, and a stronger negative correlation with Antizionist Antisemitism, *r*_s_ =  − 0.28. Similar correlations between self-identified ideological position and the same measures of antisemitism have been reported in earlier studies carried out in the same national context^18^, indicating that the sample is likely to have been typical in this respect, at least with regard to the UK adult population.

### Confirmatory analysis

For tests of pre-registered hypotheses, see Table 4. There were no outliers, as defined above, and thus there was no need to report correlations both before and after their removal, as specified in the pre-registration document. All three hypotheses were supported, both before and after partialing out the demographic variables of age, female gender, other-than-white ethnicity, and university-level education. That is, coronavirus conspiracism, measured in the form of Coronavirus Conspiracy Suspicion, was found to be sufficiently positively correlated with all forms of antisemitism in the sample for the null hypothesis of no correlation in the wider population to be rejected in all cases at *p* < 0.001 (i.e. a much more stringent level of alpha than the pre-registered cut-off of *p* < 0.010), both before and after demographic controls. However, H3 — the hypothesis of a correlation between coronavirus conspiracism and antisemitism as expressed in relation to Israel and its supporters — was only barely supported, in that the correlation observed in the sample — while very highly statistically significant — was so weak as to hover on the threshold of reportability (although it became marginally stronger after partialing out of demographic controls, indicating that the latter variables may have masked the relationship to some extent).

**Table 4:**
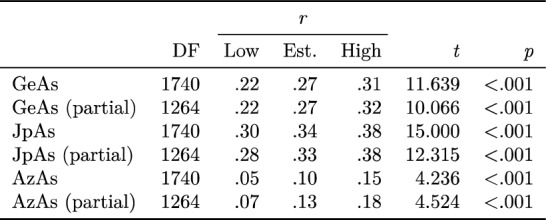
Pre-registered hypothesis tests.

Coefficients reported with 95% confidence intervals.

### Exploratory analysis

As reported above, Judeophobic Antisemitism and Antizionist Antisemitism were positively correlated, *r*(1788) = 0.40, t = 18.229, *p* < 0.001, 95% CI [0.36, 0.43]. Together with the finding of much stronger correlation between Judeophobic Antisemitism and Coronavirus Conspiracy Suspicion than between Antizionist Antisemitism and Coronavirus Conspiracy Suspicion (see above), this raised the suspicion that the correlation between Antizionist Antisemitism and Coronavirus Conspiracy Suspicion might be spurious. This possibility was investigated on an exploratory basis. Partialing out Judeophobic Antisemitism left a very weak, non-significant negative correlation between Antizionist Antisemitism and Coronavirus Conspiracy Suspicion, *r*(1739) =  − 0.04, $t$ =  − 1.544, *p* = 0.123, 95% CI [− 0.08, 0.01]. Additionally partialing out the same demographic controls employed the confirmatory analysis left virtually no correlation at all between Antizionist Antisemitism and Coronavirus Conspiracy Suspicion, *r*(1263) =  − 0.02, t =  − 0.567, *p* = 0.571, 95% CI [− 0.07, 0.04]. That is, the very weak positive correlation between Antizionist Antisemitism and Coronavirus Conspiracy Suspicion was found to be entirely accounted for by the much stronger positive correlations between Antizionist Antisemitism and Judeophobic Antisemitism and between Judeophobic Antisemitism and Coronavirus Conspiracy Suspicion, together with various mutual correlations with demographic variables.

The descriptive statistics presented above also revealed a moderate positive correlation between ideological self-placement to the left of the centre and Antizionist Antisemitism, as well as a moderate negative correlation between such ideological self-placement and both Coronavirus Conspiracy Suspicion and Judeophobic Antisemitism. Such mutual associations could potentially mask any association between Coronavirus Conspiracy Suspicion and Antizionist Antisemitism, and exaggerate any association between Coronavirus Conspiracy Suspicion and Judeophobic Antisemitism. Accordingly, it was found that controlling for self-placement to the left of the centre in addition to demographic variables weakened the correlation between Coronavirus Conspiracy Suspicion and Judeophobic Antisemitism, *r*(999) = 0.29, *t* = 9.638, *p* < 0.001, 95% CI [0.23, 0.35], while slightly strengthening the correlation between Coronavirus Conspiracy Suspicion and Antizionist Antisemitism, *r*(999) = 0.14, *t* = 4.488, *p* < 0.001, 95% CI [0.08, 0.20] (it made practically no difference to the correlation between Coronavirus Conspiracy Suspicion and Generalised Antisemitism, *r*(999) = 0.25, *t* = 8.224, *p* < 0.001, 95% CI [0.19, 0.31]). However, further controling for Judeophobic Antisemitism had the effect of completely destroying the correlation between Antizionist Antisemitism and Coronavirus Conspiracy Suspicion, *r*(998) = 0.00, *t *= 0.091, *p* = 0.928, 95% CI [− 0.06, 0.06]. That is, the moderate association between Judeophobic Antisemitism and Coronavirus Conspiracy Suspicion indeed appears to have been slightly exaggerated, and the weak association between Antizionist Antisemitism and Coronavirus Conspiracy Suspicion slightly masked, by mutual correlation with ideological position. However, even once this is controlled for, the positive relationship between Antizionist Antisemitism and Coronavirus Conspiracy Suspicion still appears to be entirely accounted for by mutual correlation with Judeophobic Antisemitism.

## Discussion

Unlike the sole previous psychological study investigating antisemitism and coronavirus conspiracism, the current study has used a representative sample, and, rather than using responses on an unconventional scale to a single untested questionnaire item which conflated antisemitism and coronavirus conspiracism, it has measured these two traits separately, using validated instruments that have also been used in other peer-reviewed studies, and it has calculated coefficients of correlation between these variables both before and after applying demographic and ideological controls. Because of the particular instrument used to measure antisemitism, it has been possible to calculate coefficients of correlation between coronavirus conspiracism and both ‘new’ and ‘old’ forms of antisemitism separately, and to establish whether correlation with either might be explained by mutual correlation with the other.

The confirmatory analysis presented here suggests that a positive correlation between Coronavirus Conspiracy Suspicion and all forms of antisemitism exists within the general UK population. However, that analysis does not sugest that all forms of antisemitism are equally related to coronavirus conspiracism, as the correlation with Judeophobic Antisemitism appears much stronger than that with Antizionist Antisemitism, and slightly stronger than that with Generalised Antisemitism (which is the overlap between the two specific forms of antisemitism). Moreover, exploratory analysis finds that the correlation between Antizionist Antisemitism and Coronavirus Conspiracy Suspicion appears to be explained by mutual correlation with Judeophobic Antisemitism.

To put this in layperson’s terms, while a person with stronger antisemitic attitudes as expressed in relation to Israel and its supporters was found to be likely to have (very slightly) higher levels of conspiracy suspicion with regard to the novel coronavirus, this was only because such a person was likely *also* to have stronger antisemitic attitudes as expressed in relation to Jews identified as Jews, and because people who hold *those* attitudes more strongly were found to be likely to have (much more notably) higher levels of conspiracy suspicion with regard to the novel coronavirus. Here, a possible parallel may be drawn with the earlier finding that Antizionist Antisemitism correlates only weakly with conspiracy beliefs relating to personal wellbeing, but is correlated much more strongly with conspiracy beliefs relating to government malfeasance, while Judeophobic Antisemitism correlates much more strongly with conspiracy beliefs relating to personal wellbeing^18^. The measure of conspiracy belief used in the latter study was the Generic Conspiracist Belief scale, whose ‘personal wellbeing’ factor includes three conspiracy theories, one involving viruses and/or diseases, and one involving experimental new drugs^72^, and thus has a great deal of overlap with the measure of Coronavirus Conspiracy Suspicion employed in the current study. A plausible interpretation of these parallel findings might be that antisemitism expressed in relation to Jews identified as Jews is more personal in character than antisemitism expressed in relation to Israel and its supporters, and that medical conspiracy theories such as might relate to the novel coronavirus, being more directly personal, might for that reason tend to appeal to the same people.

Given this argument, it may be worth noting that correlations between Coronavirus Conspiracy Suspicion and dummy variables representing voting history were at best weak, perhaps indicating that the pandemic was simply not perceived as a party-political issue. This contrasts with a recent US-based study’s finding that COVID-19 conspiracy beliefs were among those conspiracy beliefs that correlate most strongly with political partisanship^68^. Although attitudes to the novel coronavirus became heavily politicised in some national contexts, including the US^73^, this was not the case in the UK, where policies related to vaccination and mask-wearing were supported both by the right-wing government of the day and by its left-wing opposition, perhaps due to greater political trust^74^: for example, Conservative MP Andrew Bridgen’s characterisation of mass vaccination as ‘the biggest crime against humanity since the Holocaust’ placed him at odds with the rest of his party, and led to his permanent expulsion from it^75^. Here, we should also note our finding that Coronavirus Conspiracy Suspicion was more strongly correlated with self-declared ideological position than with voting history, even though that mutual correlation still appeared to account for only a small part of the correlation between Coronavirus Conspiracy Suspicion and Judeophobic Antisemitism.

It is important not to draw from these findings the implication that the circulation of conspiracy beliefs involving the pandemic will have led to an increase in anti-Jewish sentiment. Scholars have persuasively argued that conspiracy beliefs are not attributable to short-term exposure to conspiracist discourse, being ‘the product of deep-seated motivations (e.g., identities, ideologies)' ^76^. Thus, it is entirely plausible that those who came to harbour conspiracy suspicions with regard to the pandemic did so because of pre-existing ideological and psychological motivations disposing them not only towards endorsement of these conspiracy theories, but also towards antisemitism. On the evidence of this study, however, these motivations (if they exist) do not appear to have been captured by the demographic, ideological, or political variables studies here, despite the observed association between self-placement otherwise than on the left of the subjective ideological spectrum and both Judeophobic Antisemitism and Coronavirus Conspiracy Suspicion. Thus, the finding of a statistical association between antisemitism and coronavirus conspiracism in the general UK population remains important because it supports the view that the apparent relationship discerned by previous studies of activist and social media discourse may not have been limited to those two spheres, and also because it does not seem to be explicable in terms of mutual correlation with standard demographic, political, and ideological variables.

## Limitations

Because this study is purely cross-sectional, it cannot be taken to support or refute causal hypotheses, such as the hypothesis that promotion of conspiracy theories may contribute to antisemitism. Moreover, data collection fell several months after the UK’s last coronavirus lockdown, which came to an end on 19 July 2021, but very shortly after the UK government’s introduction of compulsory mask-wearing in many indoor settings^77^, and it would appear plausible that levels and correlates of coronavirus conspiracism may have varied over time and in response to such developments as these^78^. In addition, the data collection instrument did not measure coronavirus-related conspiracy beliefs, but only coronavirus-related conspiracy suspicions, and, while conspiracy suspicions have been found to be a good proxy for conspiracy beliefs^65^, the two are not necessarily identical. Lastly, it has been argued that scales eliciting endorsement or rejection of antisemitic attitudes are likely to be affected by social desirability bias, and thus to underestimate antisemitism among highly-educated people who are better able to anticipate how their responses might be interpreted^79^.

## Scope for further work

Future work investigating the association of antisemitism with apparently unrelated conspiracy theories will be necessary in order to identify underlying factors potentially explaining such association, possibly by employing more nuanced measures of ideology such as populism or authoritarianism^18,68^ or perhaps by exploring the role of personality traits, including dark personality traits^68^. A further avenue to be explored is the possibility that antisemitism may correlate with belief in non-antisemitic conspiracy theories because conspiracy theories form a mutually reinforcing network of beliefs^80^: the conspiratorial character of antisemitism has been observed in many previous studies (see above), such that an argument can be made that antisemitism could itself to some extent be considered to constitute a conspiracy theory. Finally, it may be necessary to test for any possible relationship between conspiracism and further forms of racial, ethno-religious, or national prejudice, such as anti-Black, anti-Muslim, and anti-Chinese bigotry, in order to establish the extent to which antisemitism is unusual among or typical of such forms of prejudice in its relationship, either to coronavirus conspiracism in particular, or to conspiracism in general.

## Technical note

All analyses were carried out in R v. 4.2.3^81^, with use of the *pwr* package, v. 1.3–0^82^ for power analysis and the *psych* package, v. 2.2.3^83^ for partial correlations and statistical inference.

### Supplementary Information

Supplementary Information.

## Data Availability

The datasets generated and analysed during the current study, as well as the pre-registration document and replication code for the study itself, are available in the Open Science Framework repository, https://osf.io/yu4ar/.

## References

[1] Ball K, Lawson W, Alim T (2013). Medical mistrust, conspiracy beliefs, and HIV-related behaviour among African Americans. J. Psychol. Behav. Sci..

[2] Jolley D, Douglas KM (2014). The effects of anti-vaccine conspiracy theories on vaccination intentions. PLoS ONE.

[3] Lyons B, Merola V, Reifler J (2019). Not just asking questions: Effects of implicit and explicit conspiracy information about vaccines and genetic modification. Health Commun..

[4] Chen L, Zhang Y, Young R, Wu X, Zhu G (2020). Effects of vaccine-related conspiracy theories on chinese young adults’ perceptions of the HPV vaccine: An experimental study. Health Commun..

[5] Enders AM, Uscinski J, Klofstad C, Stoler J (2022). On the relationship between conspiracy theory beliefs, misinformation, and vaccine hesitancy. PLoS ONE.

[6] Cohn N (1967). Warrant for Genocide: The Myth of the Jewish World-Conspiracy and the Protocols of the Elders of Zion.

[7] Billig M (1978). Fascists: A Social Psychological View of the National Front.

[8] Barkun M (2003). A Culture of Conspiracy: Apocalyptic Visions in Contemporary America.

[9] Byford J (2011). Conspiracy Theories: A Critical Introduction.

[10] Imhoff R, Bruder M (2014). Speaking (un-) truth to power: Conspiracy mentality as a generalised political attitude. Eur. J. Pers..

[11] Nefes TS (2015). Understanding anti-semitic rhetoric in Turkey through the Sèvres syndrome. Turk. Stud..

[12] Imhoff R, Lamberty P (2018). How paranoid are conspiracy believers? Toward a more fine-grained understanding of the connect and disconnect between paranoia and belief in conspiracy theories. Eur. J. Soc. Psychol..

[13] Rich D (2017). The Left’s Jewish Problem: Jeremy Corbyn, Israel, and Anti-Semitism.

[14] Douglas KM, Uscinski JE, Sutton RM, Cichocka A, Nefes T, Ang CS (2019). Understanding conspiracy theories. Adv. Pol. Psychol..

[15] Allington D, Joshi T (2020). "What others dare not say": An antisemitic conspiracy fantasy and its YouTube audience. J. Contem. Antisem..

[16] Allington D, Buarque BL, Flores DB (2021). Antisemitic conspiracy fantasy in the age of digital media: Three “conspiracy theorists” and their YouTube audiences. Lang. Lit..

[17] Schuller, S. World conspiracy literature and antisemitism. *TRANSIT* **13**, 194–206 (2021). https://transit.berkeley.edu/2021/schuller-conspiracyliterature/.

[18] Allington D, Hirsh D, Katz L (2023). Antisemitism is predicted by anti-hierarchical aggression, totalitarianism, and belief in malevolent global conspiracies. Hum. Soc. Sci. Commun..

[19] Freeman D, Waite F, Rosebrock L, Petit A, Causier C, East A (2020). Coronavirus conspiracy beliefs, mistrust, and compliance with government guidelines in England. Psychol. Med..

[20] Mahmood, B. *One fifth of English People in Study Blame Jews or Muslims for COVID-19. Newsweek* (2020). https://www.newsweek.com/covid-19-conspiracy-theories-england-1505899.

[21] Brown, M. *Is there any Truth Behind the Covid-19 Conspiracy Theories? Was Covid-19 Created in a Lab, Spread by 5G Masts, or Does it all Come Back to Bill Gates? Inside the Insidious World of Conspiracy Theories. Telegraph* (2020). https://www.telegraph.co.uk/news/0/truth-behind-covid-19-conspiracy-theories/.

[22] Spiegelhalter, D., Masters, A. *Do People Believe Covid Myths? Guardian* (2020). https://www.theguardian.com/theobserver/commentisfree/2021/may/16/do-people-believe-covid-myths.

[23] Moore, J. *Is it any Wonder Some People Believe in 5G Conspiracy Theories after the Leaders we’ve had? MORE Than one in Every 16 People Still buy into the 5G Coronavirus Rubbish. So There is Probably One of your Neighbours Who’s Part of the Club, maybe a Member of your Extended Family. It’s a Scary Thought. Independent* (2020). https://www.independent.co.uk/voices/5g-coronavirus-conspiracy-theory-chinese-politics-a9528896.html.

[24] McManus S, D’Ardenne J, Wessely S (2020). Covid conspiracies: Misleading evidence can be more damaging than no evidence at all. Psychol. Med..

[25] Sutton RM, Douglas KM (2020). Agreeing to disagree: Reports of the popularity of Covid-19 conspiracy theories are greatly exaggerated. Psychol. Med..

[26] Walker, P. *Jewish Group and MPs urge GB News to Stop Indulging Conspiracy Theories: Fears Antisemitic Tropes are Being Spread after Host Neil Oliver Discusses Plan to Impose ’One-World Government’. Guardian* (2023). https://www.theguardian.com/media/2023/feb/08/jewish-groups-urge-gb-news-to-stop-indulging-conspiracy-theories.

[27] Baider F (2022). Covert hate speech, conspiracy theory and anti-semitism: Linguistic analysis versus legal judgement. Int. J. Sem. Law Rev..

[28] Gunz, H., Schaller, I. Antisemitic narratives on YouTube and Telegram as part of conspiracy beliefs about COVID-19. In: Hübscher M, Mering S von, editors. *Antisemitism on Social Media*, London, pp. 129–49 (2022) DOI: 10.4324/9781003200499-9.

[29] Giry J, Butter M, Knight P (2023). Covid conspiracy theories in France. Covid Conspiracy Theories in Global Perspective.

[30] Gerstenfeld, M. Anti-Jewish Coronavirus Conspiracy Theories in Historical Context. In The COVID-19 Crisis: Impact and Implications (ed E. Karsh) 41–45 (Begin-Sadat Center for Strategic Studies, 2020). http://www.jstor.org/stable/resrep26356.12.

[31] Garner, G., McGrann, M., Klug, D., Kranson, R., Yoder, M.M. *The Relationship Between Antisemitism and COVID-19 Conspiracy on Twitter* (2023) DOI: 10.33767/osf.io/syrac.

[32] Perry SL, Whitehead AL, Grubbs JB (2020). Prejudice and pandemic in the promised land: How white Christian nationalism shapes Americans’ racist and xenophobic views of COVID-19. Ethn. Racial Stud..

[33] Awan I, Carter P, Sutch H, Lally H (2023). Online extremism and islamophobic language and sentiment when discussing the COVID-19 pandemic and misinformation on Twitter. Ethn. Racial Stud..

[34] Améry J, Améry J, Gallner M (1969). The New Left’s approach to ’Zionism’. Essays on Antisemitism, Anti-Zionism, and the Left.

[35] Patterns of Prejudice (1970). “Anti-zionists” and antisemites. Patterns of Prejud..

[36] Billig M (1984). Anti-Jewish themes and the British far left — i. Patterns of Prejud..

[37] Billig M (1984). Anti-Jewish themes and the British far left — ii. Patterns Prejud..

[38] Taguieff P-A (2004). Rising from the Muck: The New Anti-Semitism in Europe.

[39] Wistrich, R. *The politics of ressentiment: Israel, Jews, and the German media. Jerusalem: Vidal Sassoon International Center for the Study of Antisemitism.* https://sicsa.huji.ac.il/sites/default/files/sicsa/files/acta23.pdf (2004).

[40] Fine R, Spencer P (2017). Antisemitism and the Left: On the Return of the Jewish Question.

[41] Hirsh D (2017). Contemporary Left Antisemitism.

[42] Klug B (2012). Interrogating ’new anti-Semitism’. Ethn. Racial Stud..

[43] Frindte W, Wettig S, Wammetsberger D (2005). Old and new anti-semitic attitudes in the context of authoritarianism and social dominance orientation: Two studies in Germany. Peace Confl. J. Peace Psychol..

[44] Kaplan EH, Small CA (2006). Anti-Israel sentiment predicts anti-semitism in Europe. J. Confl. Resol..

[45] Baum SK, Nakazawa M (2007). Anti-semitism versus anti-Israeli sentiment. J. Relig. Soc..

[46] Cohen F, Jussim L, Harber KD, Bhasin G (2009). Modern anti-semitism and anti-Israeli attitudes. J. Pers. Soc. Psychol..

[47] Jaspal R (2013). Anti-semitism and anti-Zionism in Iran. Isr. Aff..

[48] Beattie P (2017). Anti-semitism and opposition to Israeli government policies: The roles of prejudice and information. Ethn. Racial Stud..

[49] Staetsky LD (2017). Antisemitism in Contemporary Great Britain: A Study of Attitudes Towards Jews and Israel.

[50] Staetsky LD (2020). The left, the right, christians, muslims, and detractors of Israel: Who is antisemitic in Great Britain in the early 21st century?. Contemp. Jew..

[51] Allington D, Hirsh D, Katz L (2022). The generalised antisemitism (GeAs) scale: Validity and factor structure. J. Contem. Antisem..

[52] Hirsh D, Miller H (2022). Durban antizionism: Its sources, its impact, and its relation to older anti-jewish ideologies. J. Contem. Antisem..

[53] IHRA. *Working Definition of Antisemitism* (2016). https://www.holocaustremembrance.com/resources/working-definitions-charters/working-definition-antisemitism.

[54] IHRA. *Information on Endorsement and Adoption of the IHRA Working Definition of Antisemitism* (2020). https://www.holocaustremembrance.com/resources/working-definitions-charters/working-definition-antisemitism/adoption-endorsement.

[55] Anziska, S., Assmann, A., Confino, A., Dische-Becker, E., Feldman, D., Goldberg, A., et al. *The Jerusalem Declaration on Antisemitism* (2021). https://jerusalemdeclaration.org/.

[56] Back, A., Lopezrevoredo, A., Biale, D., Schraub, D., Penslar, D., Winston, D.H., et al. The Nexus Document (2021). https://israelandantisemitism.com/the-nexus-document/.

[57] Harrison, B., Klaff, L. In Defence of the IHRA Definition. Fathom 2020; January. https://fathomjournal.org/in-defence-of-the-ihra-definition/.

[58] Nelson C (2021). Accommodating the New Antisemitism: A Critique of the ’Jerusalem Declaration’.

[59] Bogle JJJ (2022). Contemporary discourses on general definitions of antisemitism: A review article. Nord. Judaistik Scand Jew. Stud..

[60] Harrison B (2022). In defense of the IHRA definition (despite its defects as a definition). J. Contem. Antisem..

[61] Penslar, D., Who’s afraid of defining antisemitism? Antisemitism Studies 6:133–45 (2022). https://www.muse.jhu.edu/article/852572.

[62] Steinberg GM (2022). The central political role of german left actors in the campaign to replace the IHRA working definition of antisemitism. J. Contem. Antisem..

[63] Wallach Y (2022). How to fight antisemitism? Lessons from the Russian revolution. Ethn. Racial. Stud..

[64] Nosek BA, Ebersole CR, DeHaven AC, Mellor DT (2018). The preregistration revolution. PNAS.

[65] Wood MJ (2017). Conspiracy suspicions as a proxy for beliefs in conspiracy theories: Implications for theory and measurement. Brit. J. Psychol..

[66] Allington D, McAndrew S, Moxham-Hall V, Duffy B (2021). Coronavirus conspiracy suspicions, general vaccine attitudes, trust, and coronavirus information source as predictors of vaccine hesitancy among UK residents during the COVID-19 pandemic. Psychol. Med..

[67] Allington D, Hirsh D, Katz L (2022). The generalised antisemitism (GeAs) scale: A questionnaire instrument for measuring antisemitism as expressed in relation both to Jews and to Israel. J. Contemp. Antisem..

[68] Uscinski J, Enders A, Diekman A, Funchion J, Klofstad C, Kuebler S (2022). The psychological and political correlates of conspiracy theory beliefs. Sci. Rep..

[69] Hersh, E., Royden, L., Antisemitic Attitudes Across the Ideological Spectrum. (2021) https://www.eitanhersh.com/uploads/7/9/7/5/7975685/hersh_royden_antisemitism_040921.pdf.

[70] van Prooijen J-W, Krouwel APM, Pollet TV (2015). Political extremism predicts belief in conspiracy theories. Soc. Psychol. Pers. Sci..

[71] Cohen J (1988). Statistical Power Analysis for the Behavioural Sciences.

[72] Brotherton R, French C, Pickering A (2013). Measuring belief in conspiracy theories: The generic conspiracist beliefs scale. Front. Psychol..

[73] Stroebe W, vanDellen MR, Jr Abakoumkin G, Schiavone WM, Agostini M (2021). Politicization of COVID-19 health-protective behaviors in the United States: Longitudinal and cross-national evidence. PLoS ONE.

[74] Weinberg J (2022). Can political trust help to explain elite policy support and public behaviour in times of crisis? Evidence from the united kingdom at the height of the 2020 coronavirus pandemic. Pol. Stud..

[75] Baker T., *Andrew Bridgen: MP Kicked out of Tory Party After Comparing COVID Vaccines to Holocaust: Mr Bridgen was Suspended from the Parliamentary Conservative Party Following his Remarks at the Start of this Year. He has now been Ejected Completely from the Party but Says he Intends to Stand Again for Election*. Sky News (2023). https://news.sky.com/story/andrew-bridgen-mp-kicked-out-of-tory-party-after-comparing-covid-vaccines-to-holocaust-12866848.

[76] Uscinski J, Enders AM, Klofstad C, Stoler J (2022). Cause and effect: On the antecedents and consequences of conspiracy theory beliefs. Curr. Opin. Psychol..

[77] IfG. *Timeline of UK Government Coronavirus Lockdowns and Measures*. March 2020 to December 2021 2021. https://www.instituteforgovernment.org.uk/sites/default/files/2022-12/timeline-coronavirus-lockdown-december-2021.pdf.

[78] Bierwiaczonek K, Gundersen AB, Kunst JR (2022). The role of conspiracy beliefs for COVID-19 health responses: A meta-analysis. Curr. Opin. Psychol..

[79] Greene, J.P., Cheng, A., Kingsbury, I., *Education and anti-semitism. University of Arkansas: Education Reform Faculty and Graduate Students Publications* (2021) https://scholarworks.uark.edu/edrepub/121/.

[80] Williams MN, Marques MD, Hill SR, Kerr JR, Ling M (2022). Why are beliefs in different conspiracy theories positively correlated across individuals? Testing monological network versus unidimensional factor model explanations. Br. J. Soc. Psychol..

[81] R Core Team., *R: A Language and Environment for Statistical Computing*. Vienna, Austria: R Foundation for Statistical Computing (2023). https://www.R-project.org/.

[82] Champely, S., Pwr: Basic Functions for Power Analysis (2020). https://CRAN.R-project.org/package=pwr.

[83] Revelle, W., *Psych: Procedures for Psychological, Psychometric, and Personality Research. Evanston*. Northwestern University (2022). https://CRAN.R-project.org/package=psych.

