# Supplementary material for: Correlation between coronavirus conspiracism and antisemitism: a cross-sectional study in the United Kingdom
Source: Sci Rep. 2023 Dec 5;13:21104. doi: 10.1038/s41598-023-41794-y (PMC10697971; doi:10.1038/s41598-023-41794-y)
Supplement: Supplementary file 1 — Supplementary Information. [file 41598_2023_41794_MOESM1_ESM.pdf]

# Correlation between coronavirus conspiracism and antisemitism: a cross-sectional study in the United Kingdom (Appendix)

Daniel Allington David Hirsh Louise Katz

2023-05-10

## Coronavirus Conspiracy Suspicion (CCS)

To what extent, if at all, do you agree or disagree with the following statements?

- The real truth about coronavirus is being kept from the public
- People need to wake up and start asking questions about coronavirus
- Legitimate questions about coronavirus are being suppressed by the government, the media, and academia
- Reporters, scientists, and government officials are involved in a conspiracy to cover up important information about coronavirus
- An impartial, independent investigation of coronavirus would show once and for all that we've been lied to on a massive scale

Answer options: Strongly agree, Tend to agree, Neither agree nor disagree, Tend to disagree, Strongly disagree [1,2]

## Generalised Antisemitism (GeAs)

### Judeophobic Antisemitism (JpAs)

Here are a number of comments that different people have made about Jewish people in Britain. For each of the following statements, please indicate how much you agree or disagree.

- Jewish people can be trusted just as much as other British people in business (R)
- Jewish people are just as loyal to Britain as other British people (R)
- I am just as open to having Jewish friends as I am to having friends from other sections of British society (R)
- Compared to other groups, Jewish people have too much power in the media

- Jewish people talk about the Holocaust just to further their political agenda
- Jewish people chase money more than other people do

## Antizionist Antisemitism (AzAs)

Here are a number of comments that different people have made about Israel. For each of the following statements, please indicate how much you agree or disagree.

- I am comfortable spending time with people who openly support Israel (R)
- Israel has a right to exist as a homeland for the Jewish people (R)
- Israel is right to defend itself against those who want to destroy it (R)
- Israel and its supporters are a bad influence on our democracy
- Israel can get away with anything because its supporters control the media
- Israel treats the Palestinians like the Nazis treated the Jews

Answer options: Strongly agree, Agree, Neither agree nor disagree, Disagree, Strongly disagree [3,4]


[1] Wood MJ. Conspiracy suspicions as a proxy for beliefs in conspiracy theories: Implications for theory and measurement. British Journal of Psychology 2017;108:507–27. https://doi.org/https://doi.org/10.1111/bjop.12231.

[2] Allington D, McAndrew S, Moxham-Hall V, Duffy B. Coronavirus conspiracy suspicions, general vaccine attitudes, trust, and coronavirus information source as predictors of vaccine hesitancy among UK residents during the COVID-19 pandemic. Psychological Medicine 2021:1–17. https://doi.org/10.1017/S0033291721001434.

[3] Allington D, Hirsh D, Katz L. The Generalised Antisemitism (GeAs) scale: A questionnaire instrument for measuring antisemitism as expressed in relation both to Jews and to Israel. Journal of Contemporary Antisemitism 2022;5:37–48. https://doi.org/10.26613/jca/5.1.99.

[4] Allington D, Hirsh D, Katz L. The Generalised Antisemitism (GeAs) scale: Validity and factor structure. Journal of Contemporary Antisemitism 2022;5:1–28. https://doi.org/10.26613/jca/5.2.113.
